# Real-Time Robust and Optimized Control of a 3D Overhead Crane System

**DOI:** 10.3390/s19153429

**Published:** 2019-08-05

**Authors:** Arash Khatamianfar, Andrey V. Savkin

**Affiliations:** School of Electrical Engineering and Telecommunications, University of New South Wales, Sydney, NSW 2052, Australia

**Keywords:** robot control, robotic systems modeling, high-gain observers for robotic systems, position sensors, computed torque control, feedforward control, motion planning, 3D overhead crane, passivity and ℒ_2_ stability, trajectory tacking

## Abstract

A new and advanced control system for three-dimensional (3D) overhead cranes is proposed in this study using state feedback control in discrete time to deliver high performance trajectory tracking with minimum load swings in high-speed motions. By adopting the independent joint control strategy, a new and simplified model is developed where the overhead crane actuators are used to design the controller, with all the nonlinear equations of motions being viewed as disturbances affecting each actuator. A feedforward control is then designed to tackle these disturbances via computed torque control technique. A new load swing control is designed along with a new motion planning scheme to robustly minimize load swings as well as allowing fast load transportation without violating system’s constraints through updating reference trolley accelerations. The stability and performance analysis of the proposed discrete-time control system are demonstrated and validated analytically and practically.

## 1. Introduction

Overhead cranes have been one of the core pieces of equipment in the transportation industry, which present many challenges in automating their control operation due to their highly nonlinear dynamics and underactuated nature (more control variables than the number of control inputs). Moving the load with high precision in minimum time for efficiency as well as suppressing load swings for safety are the key control requirements. High-speed load motion would usually require high-speed load hoisting during trolley acceleration, which intensifies load swings and complicates the control operation. To address these problems, many studies have been conducted since the past couple of decades. From early works in this field in the 1980s, for instance, a minimum-time control problem was solved in [1] for swing-free velocity profiles. In [2], a feedback control based on swing dynamics of the load was proposed, and in [3], root locus method was used to design a feedback control law for an overhead crane. Nearly 20 years later, the full nonlinear equations of motion for a three-dimensional (3D) overhead crane were derived in spherical coordinates and Cartesian coordinates in [4,5], respectively, and linearization methods were used to control overhead crane motion. Similar linear model was used in [6] to control overhead crane with an observer-based controller and a dynamic inversion procedure. The authors in [7] applied a discrete-time integral sliding mode control on a non-minimal linear model of overhead crane. Discrete-time model predictive control (MPC) was utilized for a linearized model of a two-dimensional (2D) overhead crane to maintain load swings within acceptable range and avoid actuators saturation using constraint optimization nature in MPC [8]. Similar use of MPC on a linearize model of an overhead crane with virtual disturbance estimation was recently proposed in [9].

The advent of the Internet of Things (IoT) has brought some new initiatives on how to integrate smart sensors and adapt the control algorithm for controlling connected devices [10]. An intriguing investigation on the development of the low-cost sensors for overhead crane control within an industrial Internet project was proposed in [11]. This research is based on a case study on the use of proof-of-concept prototypes and the hypothesis on the application of agile product development methods, such as Wayfaring, for high precision control of overhead crane through an Industrial Internet network. Another work using IoT was proposed in [12] for overhead cranes with multibody payloads. A Functional Mock-Up Interface (FMI) was used to validate the model of the double-pendulum like dynamics as a payload of the crane, and input shaping control algorithm was applied to work with IoT device implemented on a single microcontroller.

Nonlinear control techniques have also been attempted to control overhead cranes such as a PD control design with nonlinear feedback terms to increase the coupling between gantry and payload and improve the transient behavior of the overhead carne in [13]. This category of controllers is known as energy-based coupling controllers which has been utilized in [14,15,16], and very recently in [17]. The linear-in-parameter form of overhead crane nonlinear dynamics makes it possible to use adaptive control algorithms to reduce the effect of parameter uncertainty such as those reported in [18,19]. Other nonlinear techniques have also been investigated on overhead cranes such as partial feedback linearization [20], full feedback linearization using swing angle and its rate in spherical-coordinates [21], gain scheduling [22], nonlinear switching control [23], augmented LQR with sliding control [24], discrete-time MPC with feedforward control [25], and nonlinear MPC [26,27].

Model-free control algorithms have been suggested for overhead crane as well to avoid dealing with its complex nonlinear dynamics, including the early work in [28] where a fuzzy logic controller was used to reduce the load swing with a simple PD controller for position control. In paper [29], a sliding mode controller with fuzzy tuning for the sliding surface was proposed. A full fuzzy controller was developed in [30] with an adaptive algorithm to tune the free parameters of the control system. A state-feedback controller was designed based on a three-rule Takagi-Sugeno fuzzy model of the overhead crane with a saturated input so that trajectories of the system starting from an ellipsoid would remain in it [31]. To deal with parameter variations and uncertain disturbances, a Type-2 fuzzy controller is combined with sliding mode control was proposed in [32], where a tuning parameter mechanism based on adaptive differential evolutionary algorithm was utilized for optimal control performance. Moreover, a PID-neural network controller was proposed in [33] which tunes PID gains using standard weights training algorithms. Lately, some attempts have also been made to apply visual-based feedback control using standard CCD (charge-coupled device) cameras to capture the dynamic movement of the overhead crane. For instance, in [34], visual tracking was proposed based on color histograms to track a dynamic object in a 2D overhead crane, and similar approach was adopted for a 3D overhead crane in [35] by comparing the lightest or darkest points in positioning area of a dynamic object and then computes the necessary trolley position and load swing.

One problem with the majority of the aforementioned works is that their control systems were designed without load hoisting action, i.e., hoisting rope length is considered either constant or varying very slowly. However, high-speed load hoisting is a pivotal factor in increasing efficiency in practice even though it adds to the complexity of the control process. The reason is that load swing increases significantly when the load is hoisted up rapidly during acceleration, as mentioned earlier. The initial studies addressing high-speed load hoisting was conducted in [36], in which a trajectory tracking controller was designed based on a Lyapunov technique and the full nonlinear model of overhead crane in [5]. A different approach was suggested in [37] with load-hoisting capability where load swing dynamics is coupled with trolley motion via a linear PD-type sliding surface. Similar technique was adopted in [38] using sliding mode control. Second-order sliding mode controllers were used for 3D overhead crane in [39,40] considering load hoisting. More recently, the authors of [41] proposed an adaptive fuzzy sliding mode control with hoisting ability on a 2D overhead crane where sliding surface was defined in a way to include trolley acceleration in swing dynamics so that it could provide nonlinear swing damping,. In [42], an adaptive coupling control law was proposed for 2D overhead crane to handle load hoisting control subjected to unknown load mass.

Motion planning for overhead cranes has also drawn some attention lately, which aims to find a reference trajectory to provide minimum-time motion with less swing angle while satisfying physical constraints of the overhead crane. The pioneering work in this area was developed by Lee in [43], where the motion-planning problem was solved as a kinematic problem using swing dynamics and Lyapunov stability theorem for a 2D overhead crane. The improved version of this technique for 3D overhead cranes was proposed in [44] where both support load hoisting. Most recently, some efforts have been made to develop different motion-planning algorithms such as the works in [45,46,47], although their focus was on 2D overhead crane without load hoisting.

In this article, a new and advanced control system for 3D overhead cranes is developed in discrete-time to deliver high performance control operation for overhead cranes in fully automated fashion that can provide time-efficient load transportation, high accuracy in load positioning, load swing suppression over the entire operation, and high-speed load hoisting ability, which comprises of four main parts. The first part is the main discrete-time controller, which calculates control inputs to perform trajectory tracking based on state feedback approach. Second part is a reference signal generator that provides reference trajectories similar to typical anti-swing trajectory performed by an expert crane operator [43], alongside a new motion planning scheme which takes into account overhead crane actuators’ constraints. The third part is a feedforward control action to compensate for disturbances and uncertainties and improve load positioning accuracy and robustness using computed torque control [48]. The last part is a new load swing control, inspired by [44], that modifies reference traveling and traversing accelerations to enable robust load swing suppression. The foundation of the designed control system is the so-called independent joint control strategy, adopted from the field of robot manipulator control. In this control strategy, the controller is designed of for the process actuators rather than the process itself, and all the nonlinear dynamics due to coupling between mechanical structure of the process and the actuators are modeled as disturbances affecting the actuators [48,49]. Thus, the overall control system design is significantly simplified without compromising the performance of the control operation as one of the primary contributions of this work. The resulting independent joint model facilitates the identification of physical parameters of the system with high precision that would otherwise be impossible to be measured individually [50], particularly coulomb friction forces, which are then incorporated into the proposed control system as part of pre-known disturbances. From practical perspective, discrete-time nature of the overall design eliminates the issues concerning quantization errors and sampling time choice and facilitates implementation of the control system on any industrial digital processor. This work is the complete upgrade of our previous works conducted on 2D overhead cranes [51,52] as part of a project in [53], to make it compatible with highly complex 3D overhead cranes with rigorous analytical proof on stability and robustness of the whole control system as well as several practical tests. Furthermore, our new motion planning scheme proposes a comprehensive algorithm, which in conjunction with our load swing control, guarantees time-efficient reference trajectories in real-time without violating actuators’ constraints as another major contribution of this work.

The paper is organized as follows: the derivation of the proposed dynamic model of the overhead crane is described in Section 2. Section 3 describes the proposed discrete-time control system design. Section 4 covers stability analysis which is followed by real-time motion planning scheme in Section 5. Practical results and evaluation of the proposed control system are provided in Section 6. Finally, Section 7 concludes the paper.

## 2. Three-Dimensional Overhead Crane Modeling

### 2.1. Equations of Motion

**Figure 1:**
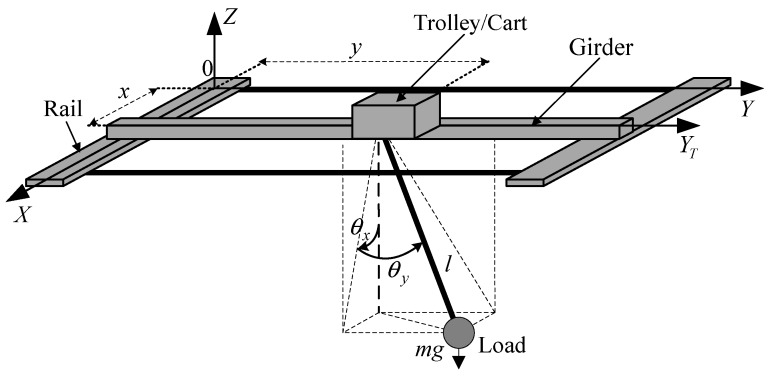
Schematic structure for a 3D overhead crane [53].

The coordinate system and schematic structure of a 3D overhead crane and its load are illustrated in Figure 1, with the following equations of motion assuming that load mass is a point mass, mass and stiffness of hoisting rope are neglected, and the connection between hoist and trolley is frictionless [5]:

(1) $$\begin{array}{l} (m_{x}+m)\ddot{x}+mlC_{\theta_{x}}C_{\theta_{y}}{\ddot{\theta }}_{x}-mlS_{\theta_{x}}S_{\theta_{y}}{\ddot{\theta }}_{y}+mS_{\theta_{x}}C_{\theta_{y}}\ddot{l}\\ +D_{x}\dot{x}+2mC_{\theta_{x}}C_{\theta_{y}}\dot{l}{\dot{\theta }}_{x}-2mS_{\theta_{x}}S_{\theta_{y}}\dot{l}{\dot{\theta }}_{y}-2mlC_{\theta_{x}}S_{\theta_{y}}{\dot{\theta }}_{x}{\dot{\theta }}_{y}\\ -mlS_{\theta_{x}}C_{\theta_{y}}{\dot{\theta }}_{x}^{2}-mlS_{\theta_{x}}C_{\theta_{y}}{\dot{\theta }}_{y}^{2}=f_{x}, \end{array}$$

(2) $$\begin{array}{l} (m_{y}+m)\ddot{y}+mlC_{\theta_{y}}{\ddot{\theta }}_{y}+mS_{\theta_{y}}\ddot{l}+D_{y}\dot{y}+2mC_{\theta_{y}}\dot{l}{\dot{\theta }}_{y}\\ -mlS_{\theta_{y}}{\dot{\theta }}_{y}^{2}=f_{y}, \end{array}$$

(3) $$\begin{array}{l} (m_{l}+m)\ddot{l}+mS_{\theta_{x}}C_{\theta_{y}}\ddot{x}+mS_{\theta_{y}}\ddot{y}+D_{l}\dot{l}-mlC_{\theta_{y}}^{2}{\dot{\theta }}_{x}^{2}-ml{\dot{\theta }}_{y}^{2}\\ -mgC_{\theta_{x}}C_{\theta_{y}}=f_{l}, \end{array}$$

(4) $$\begin{array}{l} ml^{2}C_{\theta_{y}}^{2}{\ddot{\theta }}_{x}+mlC_{\theta_{x}}C_{\theta_{y}}\ddot{x}+2mlC_{\theta_{y}}^{2}\dot{l}{\dot{\theta }}_{x}-2ml^{2}S_{\theta_{y}}C_{\theta_{y}}{\dot{\theta }}_{x}{\dot{\theta }}_{y}\\ +mglS_{\theta_{x}}C_{\theta_{y}}=0, \end{array}$$

(5) $$\begin{array}{l} ml^{2}{\ddot{\theta }}_{y}+mlC_{\theta_{y}}\ddot{y}-mlS_{\theta_{x}}S_{\theta_{y}}\ddot{x}+2ml\dot{l}{\dot{\theta }}_{y}+ml^{2}C_{\theta_{y}}S_{\theta_{y}}{\dot{\theta }}_{x}^{2}\\ +mglC_{\theta_{x}}S_{\theta_{y}}=0, \end{array}$$

where *S_θi_* and *C_θi_* denote sin*θ_i_* and cos*θ_i_*, respectively; *x* and *y* are the trolley position in *X*-axis and *Y*-axis directions, respectively; *l* is hoisting rope length; *θ_x_* and *θ_y_* are swing angles along *X*-axis and *Y*-axis directions, respectively; *m_x_*, *m_y_*, and *m_l_* are the traveling (*x*), traversing (*y*), and hoisting (*l*) components of the overhead crane mass, respectively, with each containing the equivalent masses of rotating parts such as motors and their drive trains; *D_x_*, *D_y_*, and *D_l_* denote viscous damping coefficients associated with *x*, *y*, and *l* motions, respectively; *f_x_*, *f_y_*, and *f_l_* are the driving forces in *X*-axis direction, *Y*-axis direction, and *l*-direction (hoisting), respectively; *m* is the load mass, and *g* is the gravitational acceleration.

### 2.2. Actuator Dynamics

**Figure 2:**
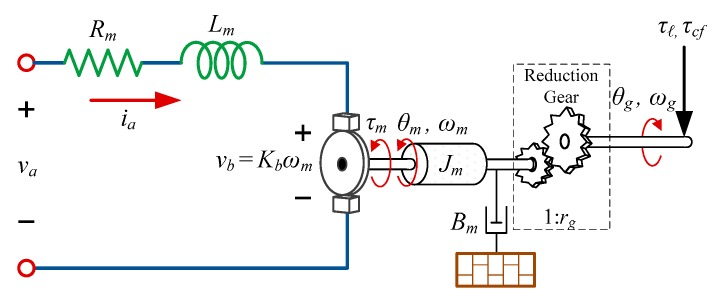
Electromechanical schematic for an armature-controlled PM DC motor.

One of the commonly-used actuators for overhead cranes is DC motors with permanent magnet (PM) and gear system as they possess high controllability and can deliver high levels of power [48,49]. The PM DC motor equation of motion as shown in Figure 2, is given as follows:

(6) $$\frac{J_{m}}{r_{g}}{\ddot{\theta }}_{g}+\frac{1}{r_{g}}(B_{m}+\frac{K_{m}K_{b}}{R_{m}}){\dot{\theta }}_{g}=\frac{K_{m}}{R_{m}}v_{a}-r_{g}\tau_{l}-\tau_{cf},$$

where θ*_g_*, ω*_g_* = $\dot{\theta }$*_g_*, and $\ddot{\theta }$*_g_* are the angular position, velocity, and acceleration of motor shaft after gearbox with gear reduction ratio *r_g_*, respectively, (i.e., θ*_g_* = *r_g_* θ*_m_* for 0 < *r_g_* < 1); *J_m_* is the total mass moment of inertia for all rotating parts of the motor; *B_m_* is the total viscous damping coefficient of bearings and shaft drag of the motor; *K_m_* is torque constant; *R_m_* is rotor winding resistance; *K_b_* is back EMF constant; *v_a_* is the applied motor voltage; *τ_ℓ_* is load torque on the motor, and *τ_cf_* is the total rotational coulomb friction force caused by interaction between motor and its connected load.

### 2.3. Independent Joint Model

A new form of dynamic model is developed here based on independent joint control strategy [48,49], with the DC motors as the main process. The nonlinear couplings, due to equations of motions of the overhead crane, are then modelled as disturbances for each actuator. A system of pulleys and belts is often used to convert angular displacement of the motor shaft to translational displacement *d*, as well as motor torque to force, i.e., *d* = *R_p_θ_g_* and *f* = *τ/R_p_* with *R_p_* as the pulley radius [48,49]. Thus, actuator equation in (6) can be written in terms of overhead crane variables for each direction of motion. By combining the overhead crane equations of motion in (1)–(5) with their corresponding actuator dynamics, the overhead crane dynamic equations are given as follows:

(7) $$J_{ex}\ddot{x}+B_{ex}\dot{x}=K_{ex}v_{ax}-f_{dx},$$

(8) $$J_{ey}\ddot{y}+B_{ey}\dot{y}=K_{ey}v_{ay}-f_{dy},$$

(9) $$J_{el}\ddot{l}+B_{el}\dot{l}=K_{el}v_{al}-f_{dl},$$

(10) $$lC_{\theta_{y}}{\ddot{\theta }}_{x}+C_{\theta_{x}}\ddot{x}+2C_{\theta_{y}}\dot{l}{\dot{\theta }}_{x}-2lS_{\theta_{y}}{\dot{\theta }}_{x}{\dot{\theta }}_{y}+gS_{\theta_{x}}=0,$$

(11) $$l{\ddot{\theta }}_{y}+C_{\theta_{y}}\ddot{y}-S_{\theta_{x}}S_{\theta_{y}}\ddot{x}+2\dot{l}{\dot{\theta }}_{y}+lC_{\theta_{y}}S_{\theta_{y}}{\dot{\theta }}_{x}^{2}+gC_{\theta_{x}}S_{\theta_{y}}=0,$$

where *J_ex_*, *J_ey_*, and *J_el_* are the total effective moment of inertia for *x*, *y*, and *l* motions, respectively, which include moment of inertia of the actuators and the corresponding crane masses, i.e., *J_ei_* = *J_mi_*/(*r_gi_ R_pi_*) + *r_gi_R_pi_m_i_* for *i* = *x*, *y*, *l*; *B_ex_*, *B_ey_*, and *B_el_* are the total damping effects related to *x*, *y*, and *l* motions, respectively, which include viscous damping of actuators and the corresponding crane damping coefficients, i.e., *B_ei_* = (*B_mi_* + (*K_mi_ K_bi_*)/*R_mi_*)/(*r_gi_ R_pi_*) + *r_gi_ R_pi_ D_i_*, and *K_ei_* = *K_mi_*/*R_mi_* for *i* = *x*, *y*, *l*; *v_ax_*, *v_ay_*, and *v_al_* are input voltages for *x*, *y*, and *l* motors, respectively; *f_dx_*, *f_dy_*, and *f_dl_* are defined as load disturbances obtained from overhead crane equations of motion in (1)–(3) without the terms that are already included in *J_ei_* and *B_ei_*, plus the coulomb friction forces acting on *x*, *y*, and *l* motions (*f_cfx_*, *f_cfy_*, and *f_cfl_*, respectively), i.e., *f_di_* = *r_gi_ R_pi_* (*f_i_* − *m_i_ d*^2^*i/dt*^2^ − *D_i_ di/dt*) + *f_cfi_* for *i* = *x*, *y*, *l*, which are given as below:

(12) $$\begin{array}{l} f_{dx}=r_{gx}R_{px}(mS_{\theta_{x}}^{2}C_{\theta_{y}}^{2}\ddot{x}+mS_{\theta_{x}}S_{\theta_{y}}C_{\theta_{y}}\ddot{y}+mS_{\theta_{x}}C_{\theta_{y}}\ddot{l}\\ \;\;\;\;\;-mlS_{\theta_{x}}C_{\theta_{y}}^{3}{\dot{\theta }}_{x}^{2}-mlS_{\theta_{x}}C_{\theta_{y}}{\dot{\theta }}_{y}^{2}-mgS_{\theta_{x}}C_{\theta_{x}}C_{\theta_{y}}^{2})+f_{cfx}, \end{array}$$

(13) $$\begin{array}{l} f_{dy}=r_{gy}R_{py}(mS_{\theta_{y}}^{2}\ddot{y}+mC_{\theta_{y}}S_{\theta_{x}}S_{\theta_{y}}\ddot{x}+mS_{\theta_{y}}\ddot{l}\\ \;\;-mlS_{\theta_{y}}C_{\theta_{y}}^{2}{\dot{\theta }}_{x}^{2}-mlS_{\theta_{y}}{\dot{\theta }}_{y}^{2}-mgC_{\theta_{y}}C_{\theta_{x}}S_{\theta_{y}})+f_{cfy}, \end{array}$$

(14) $$\begin{array}{l} f_{dl}=r_{gl}R_{pl}(m\ddot{l}+mS_{\theta_{x}}C_{\theta_{y}}\ddot{x}+mS_{\theta_{y}}\ddot{y}-mlC_{\theta_{y}}^{2}{\dot{\theta }}_{x}^{2}\\ \;\;\;\;\;-ml{\dot{\theta }}_{y}^{2}-mgC_{\theta_{x}}C_{\theta_{y}})+f_{cfl}, \end{array}$$

(15) $$f_{cfi}(v_{i})=\{\begin{array}{c} \alpha_{1i}\;\;v_{i}>0\\ -\alpha_{2i}\;\;v_{i}<0 \end{array},\;for\;i=x,\;y,\;l,$$

where (*α*_1*i*_, *α*_2*i*_) > 0 are the coulomb friction constants in positive and negative directions of motion with respect to the reference coordinate system; sgn(.) is Signum function, and *v_i_* is the translational velocity, i.e., *v_i_* = *di*/*dt* for *i* = *x*, *y*, *l* [50,54]. It should be noted that overhead crane equations of motion in (1)–(5) are simplified to obtain load disturbances in (12)–(14) knowing that *m* > 0, *l* > 0, |*θ_x_*| < *π*/2, |*θ_y_*| < *π*/2, |*C_θx_*| > 0, and |*C_θy_*| > 0. Thus, the terms *lC_θy_*$\ddot{\theta }$*_x_* and *l*$\ddot{\theta }$*_y_* in (1) and *l*$\ddot{\theta }$*_y_* in (2) can be replaced by simplified load swing dynamics in (10) and (11) [53]. The reason for this simplification is to remove swing angle accelerations from load disturbances, as they will be incorporated into feedforward control to be explained in Section 3.3.

**Remark** **1.** *Despite the underactuated nature of overhead crane, the developed independent joint model provides a set of decoupled multi–input multi–output (MIMO) linear equations as derived in (7)–(9), and simplified swing dynamics in (10) and (11), where control inputs are now the actual applied voltages to the motors rather than driving forces [55]. The first set of equations can be used for tracking control purposes, and swing dynamics can be used for load swing suppression. The effect of load swing on load positioning is reflected in the model with f_di_ as nonlinear disturbances (for i = x, y, l), which can be then compensated using another technique known as computed torque control [48]. In addition, the proposed dynamic model facilitates the estimation of model parameters including coulomb friction effects (f_cfi_), which is one of the significant forces reducing the accuracy of load positioning [38]. Further details on the parameter identification and friction model can be found in [50,53].*

### 2.4. Discrete-Time State Space Model of Overhead Crane

The proposed independent joint model in (7)–(9) can be readily transformed into discrete-time transfer function and subsequently into state space form using the integral relation between positions (*x*, *y*, *l*) and velocities (*v_x_*, *v_y_*, *v_l_*), as follows:

(16a) $$\begin{array}{l} x(z)=\frac{T_{s}}{z-1}v_{x}(z), \end{array}$$

(16b) $$\begin{array}{l} v_{x}(z)=\frac{b_{1x}}{z-a_{1x}}v_{ax}(z)-\frac{b_{d1x}}{z-a_{1x}}f_{dx}(z), \end{array}$$

(17a) $$\begin{array}{l} y(z)=\frac{T_{s}}{z-1}v_{y}(z), \end{array}$$

(17b) $$\begin{array}{l} v_{y}(z)=\frac{b_{1y}}{z-a_{1y}}v_{ay}(z)-\frac{b_{d1y}}{z-a_{1y}}f_{dy}(z), \end{array}$$

(18a) $$\begin{array}{l} l(z)=\frac{T_{s}}{z-1}v_{l}(z), \end{array}$$

(18b) $$\begin{array}{l} v_{l}(z)=\frac{b_{1l}}{z-a_{1l}}v_{al}(z)-\frac{b_{d1l}}{z-a_{1l}}f_{dl}(z), \end{array}$$

where *T_s_* is the sampling time, and (*a*_1*i*_, *b*_1*i*_, *b_d_*_1*i*_) > 0 for *i* = *x*, *y*, *l* are discrete-time transfer functions coefficients, which are obtained using the zero-order-hold (ZOH) equivalent of the first-order continuous-time transfer function [56]:

(19) $$a_{1i}=e^{-\frac{B_{ei}T_{s}}{J_{ei}}},\;\;b_{1i}=\frac{K_{ei}}{B_{ei}}(1-e^{-\frac{B_{ei}T_{s}}{J_{ei}}}),\;\;b_{d1i}=\frac{1}{B_{ei}}(1-e^{-\frac{B_{ei}T_{s}}{J_{ei}}}).$$

The proposed state space representation of the system is obtained as below when state variables are selected as ***x***(*k*) = [*x*(*k*) *v_x_*(*k*) *y*(*k*) *v_y_*(*k*) *l*(*k*) *v_l_*(*k*)]*^T^* with the output vector as ***y***(*k*) = [*x*(*k*) *y*(*k*) *l*(*k*)]*^T^* (bold notations in lowercase letters are used for vector variables):

(20) $$\begin{array}{l} x(k+1)=Ax(k)\;+\;Bu(k)+\;W_{d}f_{d}(k),\\ y(k)=Cx(k), \end{array}$$

where ***u***(*k*) = [*v_ax_*(*k*) *v_ay_*(*k*) *v_al_*(*k*)]*^T^* is the control input vector; ***f****_d_*(*k*) = [*f_dx_*(*k*) *f_dy_*(*k*) *f_dl_*(*k*)]*^T^* is the vector of input disturbances; *A* = *BlockDiag*{*A_x_*, *A_y_*, *A_l_*} is the system matrix; *B* = *BlockDiag*{*B_x_*, *B_y_*, *B_l_*} is the control input matrix; *W_d_* = *BlockDiag*{*W_dx_*, *W_dy_*, *W_dl_*} is the input disturbance matrix, and *C* = *BlockDiag*{*C_x_*, *C_y_*, *C_l_*} is the output matrix with inner matrices given as follows, (*BlockDiag*{.} and *Diag*{.} denote the block-diagonal matrix and diagonal matrix, respectively):

(21) $$\begin{array}{l} A_{i}=\left[\begin{array}{cc} 1 & T_{s}\\ 0 & a_{1i} \end{array}\right],\;\;B_{i}=\left[\begin{array}{c} 0\\ b_{1i} \end{array}\right],\;\;W_{di}=\left[\begin{array}{c} 0\\ -b_{d1i} \end{array}\right],\;\;C_{i}=\left[\begin{array}{cc} 1 & \;0 \end{array}\right]\;,\\ \mathrm{for}\;\;i=x,\;y,\;l. \end{array}$$

## 3. Configuration of the Proposed Control System

**Figure 3:**
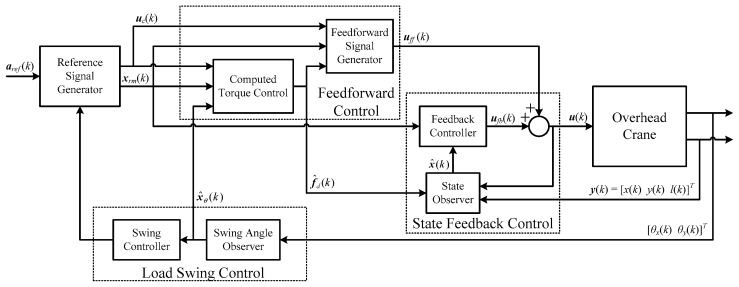
Configuration of the proposed control system for 3D overhead crane [53].

To achieve high performance control operation in 3D overhead cranes, the designed control system is constructed as shown in Figure 3 consisting of four main parts:

(1) State feedback control, which provides servo control operation for trajectory tracking control purposes along with state observer to provide estimation of states variables from position sensor measurements and attenuate the impact of measurement noises.
(2) Reference signal generator, which supplies reference state trajectory profiles considering the physical limitations of the actuators admissible torques and speeds, and overhead crane workspace, alongside a new motion planning scheme.
(3) Feedforward control, which is designed to act as a compensator by generating the desired output trajectory from system model and applying it in the feedforward path to reduce the effects of nonlinear disturbances and improve the accuracy of trajectory tracking.
(4) Load swing control, which is designed to damp the load swings by modifying reference traveling and traversing accelerations using high-gain observer, as will be explained in Section 4.1.

Modifying the reference accelerations would cause deviation in the reference position and velocity trajectories. Thus, a new motion planning scheme is developed in Section 5 as part of reference signal generation, which allows load swing suppression throughout the trajectory as well as fixing the changes in the reference position and velocity for traveling and traversing motions.

### 3.1. State Feedback Control and State Observer

The discrete-time control law for generating control input voltages in overhead crane control system using state feedback approach is given as follows:

(22) $$u(k)=u_{fb}(k)+u_{ff}(k)=K(x_{rm}(k)-\hat{x}(k))+u_{ff}(k),$$

where *K* = *BlockDiag*{*K_x_*, *K_y_*, *K_l_*} is feedback gain with *K_i_* = [*k*_1*i*_ *k*_2*i*_] for *i* = *x*, *y*, *l*; ***x****_rm_* (*k*) = [***x****_rmx_*(*k*) ***x****_rmy_*(*k*) ***x****_rml_*(*k*)]*^T^* = [*x_ref_* (*k*) *v_xref_* (*k*) *y_ref_* (*k*) *v_yref_* (*k*) *l_ref_* (*k*) *v_lref_* (*k*)]*^T^* is the reference trajectory vector provided by reference signal generator; $\hat{x}$(*k*) is the estimate of states; ***u****_fb_*(*k*) is the feedback signal aiming to reduce the error between the reference state trajectories and the system states, and ***u****_ff_*(*k*) = [*u_ffx_*(*k*) *u_ffy_*(*k*) *u_ffl_*(*k*)]*^T^* is the feedforward control signal. Since full state measurement is not available, the estimation of system states $\hat{x}$(*k*) is used in the control law by using the following dynamic state observer:

(23) $$\hat{x}(k+1)=A\hat{x}(k)\;+\;Bu(k)+\;W_{d}{\hat{f}}_{d}(k)+L(y(k)-C\hat{x}(k)),$$

where *L* = *BlockDiag*{*L_x_*, *L_y_*, *L_l_*} is the observer gain with *L_i_* = [*l*_1*i*_ *l*_2*i*_]*^T^* for *i* = *x*, *y*, *l*; and $\hat{f}$*_d_* (*k*) = [$\hat{f}$*_dx_*(*k*) $\hat{f}$*_dy_*(*k*) $\hat{f}$*_dl_*(*k*)]*^T^* is the computed load disturbance vector calculated by using (12)–(15).

### 3.2. Reference Signal Generator

Reference trajectories for traveling, traversing and hoisting positions and velocities (***x****_rm_*) are generated via discrete-time integration of the modified reference accelerations using the following state space reference model:

(24) $$x_{rm}(k+1)=A_{m}x_{rm}(k)\;+\;B_{m}u_{c}(k),$$

where ***u****_c_*(*k*) = [*u_cx_*(*k*) *u_cy_*(*k*) *u_cl_*(*k*)]*^T^* is the command signal to reference model given by modified reference traveling, traversing, and hoisting accelerations, i.e., *u_cx_*(*k*) = *a_xref_*(*k*) + *a_x_corr_*(*k*), *u_cy_*(*k*) = *a_yref_*(*k*) + *a_y_corr_*(*k*), and *u_cl_*(*k*) = *a_lref_*(*k*) + *a_l_corr_*(*k*) respectively; *A_m_* = *BlockDiag*{*A_mx_*, *A_my_*, *A_ml_*} and *B_m_* = *BlockDiag*{*B_mx_*, *B_my_*, *B_ml_*} are system and input matrices for reference model, respectively, with inner matrices as follows:

(25) $$A_{mi}=\left[\begin{array}{cc} 1 & T_{s}\\ 0 & 1 \end{array}\right],\;\;B_{mi}=\left[\begin{array}{c} 0\\ T_{s} \end{array}\right],\;\mathrm{for}\;i=x,\;y,\;l.$$

The correction terms *a_x_corr_* and *a_y_corr_* to the reference trolley accelerations *a_xref_* and *a_yref_* are calculated by load swing control in accordance with the motion planning scheme to robustly suppress load swings, and *a_l_corr_* may be needed to avoid violating actuators’ constraints as will be described in Section 4 and Section 5.

### 3.3. Feedforward Control

To compensate for nonlinear disturbances, feedforward signal ***u****_ff_* is included in the proposed discrete-time control law which is calculated using an inverse dynamic technique known as computed torque control [48], in which the ideal torques are computed using equations of motion and pre-known reference trajectories. The same concept can be applied here to obtain the ideal motor voltages. Thus, by using the discrete-time dynamic model of overhead crane derived in (16b)–(18b) and reference velocities (*v_xref_*, *v_yref_*, *v_lref_*), ***u****_ff_* can be given as follows:

(26) $$b_{1i}u_{ffi}(k)=v_{iref}(k+1)-a_{1i}v_{iref}(k)+b_{d1i}{\hat{f}}_{di}(k),\;\;\mathrm{for}\;\;i=x,\;y,\;l.$$

Since reference velocities are generated via reference model given in (24) and (25), we have *v_iref_* (*k* + 1) = *v_iref_* (*k*) + *T_s_u_ci_* (*k*), which enables us to calculate feedforward signal by using command signal ***u****_c_* and the reference model as follows:

(27) $$u_{ff}(k)=\Phi_{ff}x_{rm}(k)+\Gamma_{ff}u_{c}(k)+\Lambda_{ff}{\hat{f}}_{d}(k),$$

where Φ*_ff_* = *BlockDiag*{Φ*_ffx_*, Φ*_ffy_*, Φ*_ffl_*}; Γ*_ff_* = *Diag*{γ*_ffx_*, γ*_ffy_*, γ*_ffl_*}; Λ*_ff_* = *Diag*{λ*_ffx_*, λ*_ffy_*, λ*_ffl_*}, with inner matrices given as below:

(28) $$\Phi_{ffi}=\left[\begin{array}{cc} 0 & \frac{1-a_{1i}}{b_{1i}} \end{array}\right],\;\;\gamma_{ffi}=\frac{T_{s}}{b_{1i}},\;\;\lambda_{ffi}=\frac{b_{d1i}}{b_{1i}},\;\;\mathrm{for}\;\;i=x,\;y,\;l.$$

It can be seen from (27) and (28) that the overhead crane nonlinear effects can be compensated through feedforward signal at each sampling time due to having computed disturbances $\hat{f}$*_d_* (*k*) as part of ***u****_ff_* (*k*). Moreover, to reflect the effects of load swings in the computation of load disturbances, the online measurements of swing angles can be used rather than the desired ones (which is of course zero). However, the sensors used for swing angles cannot measure their velocities and accelerations, which is why simplified overhead crane equations of motion were used to obtain load disturbances in (12)–(15) so that only swing angles and their velocities would be needed for computation of $\hat{f}$*_d_* (*k*).

### 3.4. Swing Angle Observer

Since only swing angles measurements are available, a high-gain observer is designed as below to estimate swing angles and their velocities, due to the fact that a high-gain observer would have a better performance when an accurate model of swing dynamics is not available [57]:

(29) $${\hat{x}}_{\theta }(k+1)=A_{O\theta }{\hat{x}}_{\theta }(k)+L_{O\theta }(y_{\theta }(k)-C_{O\theta }{\hat{x}}_{\theta }(k)),$$

where $\hat{x}$*_θ_*(*k*) is the estimate of swing angles and their velocities (θ*_x_*, $\dot{\theta }$*_x_*, θ*_y_*, $\dot{\theta }$*_y_*); *A_O__θ_* = *BlockDiag*{*A_O__θx_*, *A_O__θy_*} and *C_O__θ_* = *BlockDiag*{*C_O__θx_*, *C_O__θy_*} are system and output matrices for swing angle observer, and *L_O__θ_* = *BlockDiag*{*L_O__θx_*, *L_O__θy_*} is the swing angle observer gain with inner matrices given as below:

(30) $$\begin{array}{l} A_{O\theta_{i}}=\left[\begin{array}{cc} 1 & T_{s}\\ 0 & 1 \end{array}\right],\;C_{O\theta_{i}}=[1\;0],\;L_{O\theta_{i}}=\left[\begin{array}{c} l_{1\theta_{i}}\\ l_{2\theta_{i}} \end{array}\right],\\ l_{1\theta_{i}}=\frac{\delta_{1i}+2}{\varepsilon },\;l_{2\theta_{i}}=\frac{\varepsilon^{2}+\delta_{1i}\varepsilon +\delta_{2i}}{\varepsilon T_{s}}\;\mathrm{for}\;i=x,y, \end{array}$$

for a positive constant *ε* ≪ 1, and positive constants *δ*_1*i*_ and *δ*_2*i*_, which are chosen such that the roots of *z*^2^ + *δ*_1*i*_ *z* + *δ*_2*i*_ = 0 are located inside the unit circle for *i* = *x*, *y* [53]. In Section 6.2, the operation of the swing angle observer will be shown under practical tests conducted on the designed discrete-time control system for a 3D overhead crane.

## 4. Stability and Robustness Analysis of the Proposed Discrete-Time Control System

### 4.1. Load Swing Stability

Load swing dynamics obtained in (10) and (11) can be written in a matrix from by defining ***θ*** = [*θ_x_ θ_y_*]*^T^* as follows:

(31) $$M_{\theta }\ddot{\theta }+C_{\theta }\dot{\theta }+G_{\theta }+H_{\theta }a_{xy}=0,$$

where:

(32) $$\begin{array}{l} M_{\theta }=\left[\begin{array}{cc} lC_{\theta_{y}}^{2} & 0\\ 0 & l \end{array}\right],\;C_{\theta }=\left[\begin{array}{cc} 2C_{\theta_{y}}^{2}\dot{l}-2lS_{\theta_{y}}C_{\theta_{y}}{\dot{\theta }}_{y} & 0\\ lC_{\theta_{y}}S_{\theta_{y}}{\dot{\theta }}_{x} & 2\dot{l} \end{array}\right],\\ G_{\theta }=\left[\begin{array}{c} gS_{\theta_{x}}C_{\theta_{y}}\\ gC_{\theta_{x}}S_{\theta_{y}} \end{array}\right],\;H_{\theta }=\left[\begin{array}{cc} C_{\theta_{x}}C_{\theta_{y}} & 0\\ -S_{\theta_{x}}S_{\theta_{y}} & C_{\theta_{y}} \end{array}\right],\;a_{xy}=\left[\begin{array}{c} \ddot{x}\\ \ddot{y} \end{array}\right]. \end{array}$$

As can be seen, trolley accelerations ***a****_xy_* = [$\ddot{x}$ $\ddot{y}$]*^T^* act as the input to the swing dynamics and they determine the behavior of the swing angles. Thus, load swings can remain bounded if trolley accelerations are controlled. To achieve this, some nonlinear analysis tools are needed, naming passivity-based control and $L_{2}$ stability, using the following lemma [57].

**Lemma** **1.** *Consider a time-invariant nonlinear system $\dot{x}$ = **f**(**x**,**u**) and **y** = **h**(**x**) with **x** ∈ R^n^ as system states, **u** ∈ R^p^ as input, and **y** ∈ R^m^ as output, where functions **f**(**x**,**u**) and h(**x**) are locally Lipschitz with **f**(0,0) = **h**(0) = 0. Let V(**x**) be a continuously differentiable positive semi-definite function (called storage function). If the system is output strictly passive, i.e., **u**^T^**y** ≥ $\dot{V}$ + **y**^T^**φ**(**y**) with **y**^T^**φ**(**y**) > 0, then it is finite-gain $L_{2}$ stable and its $L_{2}$ gain (||***y***||) ($||*|{|}_{L_{2}}$ and ||∗|| denotes the $L_{2}$-norm or Euclidian norm of a vector throughout this text) is less than or equal to 1/δ if **φ**(**y**) = δ**y** for δ > 0 and **u** ∈ $L_{2}$.*

Let the storage function *V_θ_* be defined as below:

(33) $$V_{\theta }=\frac{1}{2}{\dot{\theta }}^{T}M_{\theta }\dot{\theta }+g(1-C_{\theta_{x}}C_{\theta_{y}}).$$

The first time-derivative of the storage function ${\dot{V}}_{\theta }$ is obtained by replacing *M_θ_*$\ddot{\theta }$ from (31) as follows:

(34) $${\dot{V}}_{\theta }={\dot{\theta }}^{T}(\frac{1}{2}{\dot{M}}_{\theta }-C_{\theta })\dot{\theta }-{\dot{\theta }}^{T}H_{\theta }a_{xy}.$$

Using (32), (0.5$\dot{M}$*_θ_* − *C_θ_*) can be simplified as follows:

(35) $$\frac{1}{2}{\dot{M}}_{\theta }-C_{\theta }=\left[\begin{array}{cc} -\frac{3}{2}\dot{l}C_{\theta_{y}}^{2}+lS_{\theta_{y}}C_{\theta_{y}}{\dot{\theta }}_{y} & 0\\ -lC_{\theta_{y}}S_{\theta_{y}}{\dot{\theta }}_{x} & -\frac{3}{2}\dot{l} \end{array}\right],$$

and then, by expanding and rearranging $\dot{\theta }$*^T^*(0.5$\dot{M}$*_θ_* − *C_θ_*)$\dot{\theta }$ into a matrix form we have the following:

(36) $${\dot{\theta }}^{T}(\frac{1}{2}{\dot{M}}_{\theta }-C_{\theta })\dot{\theta }=-\frac{3}{2}\dot{l}C_{\theta_{y}}^{2}{\dot{\theta }}_{x}^{2}-\frac{3}{2}\dot{l}{\dot{\theta }}_{y}^{2}=-\frac{3}{2}{\dot{\theta }}^{T}{M^{\prime }}_{\theta }\dot{\theta },$$

where *M′_θ_* = *Diag*{$\dot{l}$*C*^2^*_θy_*, $\dot{l}$}. Hence, ${\dot{V}}_{\theta }$ is simplified as follows:

(37) $${\dot{V}}_{\theta }=-\frac{3}{2}{\dot{\theta }}^{T}{M^{\prime }}_{\theta }\dot{\theta }-{\dot{\theta }}^{T}H_{\theta }a_{xy}.$$

As mentioned earlier, traveling and traversing accelerations ***a****_xy_* should be manipulated to suppress load swings. Also, the discrete-time control law in (22) is designed to compute proper motor voltages so that the position and velocity of trolley and hoisting rope length can be controlled robustly to follow the reference trajectories provided by reference accelerations (*a_xref_*, *a_yref_*, *a_lref_*). According to the principal of kinematics in mechanics [48,49], to move an object from one point to another point following a specific position and velocity trajectories within a finite period, the acceleration of the object should be a function of position and velocity profiles. Therefore, it is a true assumption that when the position and velocity of the trolley follow the reference traveling and traversing trajectories by the discrete-time controller, i.e., (*x*, *v_x_*) → (*x_ref_*, *v_xref_*) and (*y*, *v_y_*) → (*y_ref_*, *v_yref_*), the trolley accelerations will eventually follow the reference traveling accelerations, i.e., ($\ddot{x}$, $\ddot{y}$) → (*a_xref_*, *a_yref_*). Otherwise, the overhead crane would never reach the final destination the way it is designed to. Thus, reference traveling and traversing accelerations ***a****_xy_***___***_ref_* = [*a_xref_ a_yref_*]*^T^* are considered as the input in place of ***a****_xy_* in (37) as below:

(38) $${\dot{V}}_{\theta }=-\frac{3}{2}{\dot{\theta }}^{T}{M^{\prime }}_{\theta }\dot{\theta }-{\dot{\theta }}^{T}H_{\theta }a_{xy\_ref}.$$

To stabilize swing dynamics a correction term ***a****_xy_corr_* = [*a_x_corr_* *a_y_corr_*]*^T^* is added to ***a****_xy_ref_* as follows:

(39) $$u_{c\_xy}=a_{xy\_ref}+a_{xy\_corr}=a_{xy\_ref}+K_{\theta }H_{\theta }^{-1}\dot{\theta },$$

where ***a****_xy_corr_* = *K_θ_ H_θ_*^−1^$\dot{\theta }$ is the vector of correction accelerations; *K_θ_* = *Diag*{*k_θx_*, *k_θy_*} is the swing control gain, and ***u****_c_xy_* = [*u_cx_ u_cy_*]*^T^* is the updated trolley accelerations, which will be used along with reference hoisting acceleration *u_cl_* = *a_lref_* as command signal for reference model in (24) to generate reference state trajectories. It should be noted that *H_θ_* is invertible for all {| *θ_x_* |, | *θ_y_* |} ≠ {*π*/2, *π*/2} (|*| denotes the absolute value of a variable.) as its inverse is obtained using (32) as below:

(40) $$H_{\theta }^{-1}=\frac{1}{C_{\theta_{x}}C_{\theta_{y}}^{2}}\left[\begin{array}{cc} C_{\theta_{y}} & 0\\ S_{\theta_{x}}S_{\theta_{y}} & C_{\theta_{x}}C_{\theta_{y}} \end{array}\right].$$

By replacing ***a****_xy_ref_* with ***u****_c_xy_* in (38), ${\dot{V}}_{\theta }$ is given as below:

(41) $${\dot{V}}_{\theta }=-\frac{3}{2}{\dot{\theta }}^{T}{M^{\prime }}_{\theta }\dot{\theta }-{\dot{\theta }}^{T}H_{\theta }(a_{ref}+K_{\theta }H_{\theta }^{-1}\dot{\theta }).$$

We can find the upper bound of ${\dot{V}}_{\theta }$ knowing that ||*H_θ_*$|{|}_{L_{1}}$≤ 1 by rearranging the terms in the above equation, which results in the following, ($||H_{\theta }|{|}_{L_{1}}$ is the $L_{1}$-norm of matrix $H_{\theta }$ defined as $\underset{1\leq j\leq n}{\max}\sum_{i=1}^{m}\left|h_{\theta_{ij}}\right|=\underset{\forall |\theta_{x}|,|\theta_{y}|<\frac{\pi }{2}}{\max}\{(C_{\theta_{x}}C_{\theta_{y}}-S_{\theta_{x}}S_{\theta_{y}}),C_{\theta_{y}}\}=\underset{\forall |\theta_{x}|,|\theta_{y}|<\frac{\pi }{2}}{\max}\{C_{\theta_{x}+\theta_{y}},C_{\theta_{y}}\}=1.$)

(42) $$a_{xy\_ref}^{T}\dot{\theta }\geq {\dot{V}}_{\theta }+{\dot{\theta }}^{T}(\frac{3}{2}{M^{\prime }}_{\theta }+K_{\theta })\dot{\theta }.$$

It can be seen from (42) that by choosing ***a****_xy_ref_* ∈ $L_{2}$ as the input, $\dot{\theta }$ as the output, and *V_θ_* as the storage function with *φ*(***y***) = (1.5*M*′*_θ_* + *K_θ_*) $\dot{\theta }$, the swing dynamics for the 3D overhead crane will be output strictly passive if $\dot{\theta }$*^T^*(1.5*M*′*_θ_* + *K_θ_*)$\dot{\theta }$ > 0. This means (1.5*M*′*_θ_* + *K_θ_*) must be positive definite, which requires that swing control gains for *x* and *y* motions are chosen as follows:

(43) $$\{k_{\theta_{x}},\;k_{\theta_{y}}\}\geq 1.5|\dot{l}{|}_{\max}.$$

Therefore, the swing dynamics which satisfies (39) and (43) are finite-gain $L_{2}$ stable, as stated in the Lemma, with its $L_{2}$ gain less than or equal to (max{*k_θx_*, *k_θy_*} + 1.5|$\dot{l}$|_max_)^−1^ where |$\dot{l}$|_max_ is the maximum absolute value of the hoisting velocity. Furthermore, when $||\dot{\theta }|{|}_{L_{2}}$ ≤ *c*_1_ < ∞, its integration over a fixed period has bounded $L_{2}$ gain since swing angles have sinusoidal behavior, i.e., $||\theta |{|}_{L_{2}}$ ≤ *c*_2_ < ∞ for positive constants *c*_1_ and *c*_2_. Therefore, (39) is considered as the proposed load swing control law for 3D overhead crane.

**Remark** **2.** *The design requirement to have high speed motion in the 3D overhead crane as well as maintaining load swing in a small range would not be satisfied without a suppressing force being applied on swing dynamics. Due to the lack of direct damping force on the load swing, it is proven that updating the reference traveling and traversing accelerations in real-time using the measurement from load swings can provide such load swing damping effect. This would act as a virtual friction force on swing angles since the correction term is a function of swing angle velocities. Moreover, this proposed load swing control can be easily implemented in discrete-time since the correction of reference accelerations can be made at each sampling time using the discrete-time values of swing angles and their velocities provided by load swing observer in (29) and (30).*

### 4.2. Trajectory Tracking Stability

We can now show how our proposed discrete-time control law given in (22) with feedforward control in (27) can provide both stability in trajectory tracking and disturbance rejection. Let us define ***e***(*k*) = ***x****_rm_*(*k*) − ***x***(*k*) as the tracking error. By using (20) and (24), the tracking error equation is given as follows:

(44) $$e(k+1)=A_{m}x_{rm}(k)-Ax(k)+B_{m}u_{c}(k)-Bu(k)-W_{d}f_{d}(k).$$

Using (22) and (27) to substitute for control input ***u***(*k*) and feedforward signal ***u****_ff_* (*k*) in (44), respectively, we have the following:

(45) $$\begin{array}{ll} e(k+1)= & Ax(k)-(A_{m}-B\Phi_{ff})x_{rm}(k)-BKe(k)+BK\hat{e}(k)\\ & -(B_{m}-B\Gamma_{ff})u_{c}(k)+B\Lambda_{ff}{\hat{f}}_{d}(k)+W_{d}f_{d}(k), \end{array}$$

where $\hat{e}$(*k*) = ***x***(*k*) − $\hat{x}$(*k*) is the state estimation error given by (20) and (23) as below:

(46) $$\hat{e}(k+1)=(A-LC)\hat{e}(k)+W_{d}(f_{d}(k)-{\hat{f}}_{d}(k)).$$

Note that the inner matrices for system model in (21), for reference model in (25), and for feedforward signal in (28) and the rest of the matrix variables all formulated in block-diagonal form. This allows for the simplification of the tracking error equation in (45) which can then be augmented to (46) to create the following tracking error equation since *A_m_* − *B*Φ*_ff_* = *A*, *B_m_* = *B*Γ*_ff_*, and *B*Λ*_ff_*= −*W_d_*:

(47) $$\left[\begin{array}{c} e(k+1)\\ \hat{e}(k+1) \end{array}\right]=\left[\begin{array}{c} A-BK\\ \underline{0} \end{array}\begin{array}{c} BK\\ A-LC \end{array}\right]\left[\begin{array}{c} e(k)\\ \hat{e}(k) \end{array}\right]+\left[\begin{array}{c} W_{d}\\ W_{d} \end{array}\right](f_{d}(k)-{\hat{f}}_{d}(k)),$$

where 0 is a zero matrix with proper size. The equation (47) shows that the tracking error dynamics is influenced directly by the model uncertainties ($f_{d}-{\hat{f}}_{d}$). The estimation of the nonlinear disturbance ${\hat{f}}_{d}$ provided by the computed torque control limits the upper bound of uncertainties, i.e., $\left\|f_{d}-{\hat{f}}_{d}\right\|$ *≤ c*_3_ *< ∞* for a small positive constant *c*_3_. Thus, the closed-loop stability and uniformly boundedness (robustness) of the tracking and state estimation errors are guaranteed when feedback gain *K* and observer gain *L* are designed to have the eigenvalues of (*A* – *BK*) and (*A* – *LC*) inside unit circle [56], i.e., $\left|\left|e\left(k\right)\right|\right|\leq \varepsilon_{1}<\infty$ and $\left|\left|\hat{e}\left(k\right)\right|\right|\leq \varepsilon_{2}<\infty$ for small positive constants *ε*_1_, and *ε*_2_.

It should be noted that feedback signal ***u****_fb_*(*k*) in discrete-time control law in (22) is defined by the error between the reference state trajectories and the estimate of system states. Thus, by defining ***e****_c_*(*k*) = x*_rm_*(*k*) − $\hat{x}$(*k*) as the controller error, the dynamic equation of controller error can also be found similar to tracking error dynamics as follows:

(48) $$\begin{array}{ll} e_{c}(k+1)= & A_{m}x_{rm}(k)-Ax(k)+B_{m}u_{c}(k)\\ & -Bu(k)-W_{d}{\hat{f}}_{d}(k)-L(y-C\hat{x}), \end{array}$$

which can be simplified as below similar to (47) by using (22) and (27), and adding and subtracting the term *LC**x**_rm_*(*k*) to (48):

(49) $$e_{c}(k+1)=(A-BK-LC)e_{c}(k)+LCe(k).$$

It can be seen from the obtained controller error dynamics in (49) that in addition to stability condition on (*A* − *BK*) and (*A* − *LC*) for uniformly boundedness of tracking error ***e***(*k*), feedback gain matrix *K* and observer gain matrix *L* should also be chosen such that the matrix (*A* − *BK* − *LC*) have all its eigenvalues inside unit circle as well. This will guarantee to have controller error uniformly bounded, i.e., ||***e****_c_*(*k*)|| ≤ *ε*_3_ < ∞ for a small positive constant *ε*_3_.

## 5. Real-Time Motion Planning Scheme

**Figure 4:**
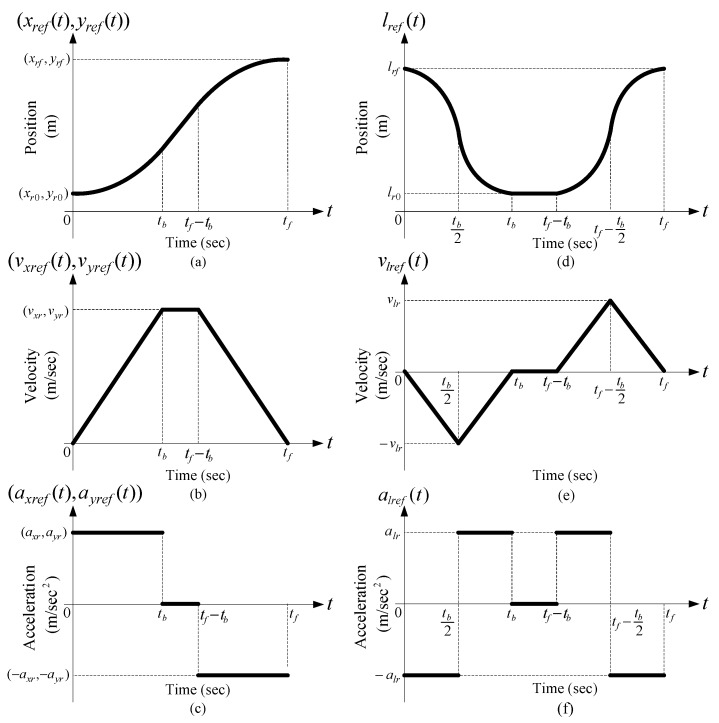
LSPB trajectories [49]: (**a**) Position; (**b**) velocity; (**c**) acceleration profiles for traveling and traversing motions; (**d**) Position; (**e**) velocity, and (**f**) acceleration profiles for hoisting motion [52].

Any motion planning is subject to constraints on the maximum permissible velocity, acceleration, and the amount of time for moving the load. In practical applications, the desired trajectory for an overhead crane is divided into three zones known as typical anti-swing trajectory [43]. In this trajectory, the overhead crane is initially accelerated up to a certain velocity while the load is lifted up. This zone is called “*accelerating zone*”. The load is then transported at that certain constant speed without any further load hoisting. This zone is called “*constant velocity zone*”. At the end, while the load gets close to the landing target, the overhead crane is decelerated to full stop while the load is hoisted down. This zone is called “*decelerating zone*”. A suitable choice for the typical anti-swing trajectories described above is the so-called “linear segments with parabolic blends” trajectories (LSBP) [49] as illustrated in Figure 4.

According to (42), it can be seen that load swing angle would grow as the trolley is accelerated if *K_θ_* is set equal to zero since $\dot{l}$ < 0 which makes *M*′*_θ_* < 0 (recall that *M*′*_θ_* = *Diag*{$\dot{l}$*C* ^2^*_θy_*, $\dot{l}$}) causing instability as swing dynamics are no longer output strictly passive. The swing angle stays the same in the constant velocity zone since $\dot{l}$ = 0. But, in decelerating zone, load swing angle would naturally decay to zero while the load is hoisted down ($\dot{l}$ > 0) even if *K_θ_* = 0. However, to have high-speed load transportation, the load should be lifted during accelerating zone and hoisted down during decelerating zone with high speed. This situation makes it difficult to damp load swings during the first two zones and could pose a real danger to the operation. Thus, high-speed load hoisting during accelerating zone is normally avoided, which lowers time efficiency.

As we elaborated in Section 4, the load swing control is designed to modify reference traveling and traversing accelerations so that the suppression of load swings during the overhead crane operation is guaranteed. However, using the modified accelerations ***u****_c_xy_* defined in (39) rather than the original ones ***a****_xy_ref_* as input to reference model in (24) would generate reference trajectories that are deviated from the original desired ones, either falling behind or overtaking. The amount of deviation depends on initial swing angle and the speed of swing reduction imposed by swing control gain *K_θ_*. This deviation causes the load to arrive at the final point with some noticeable position error.

**Figure 5:**
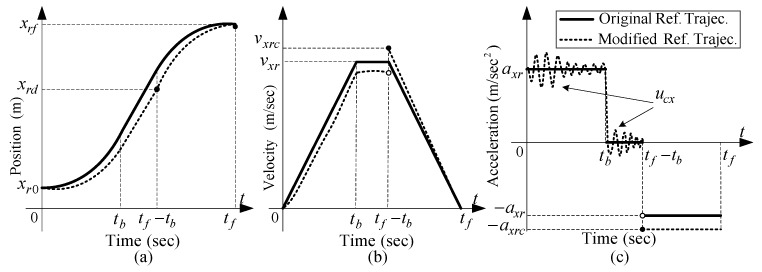
Comparison between original and modified reference traveling trajectory; (**a**) Position profile; (**b**) Velocity profile; (**c**) Acceleration profile.

To solve this problem, we can take advantage of the natural swing damping property in decelerating zone. That means the load swing control will be active during accelerating and constant-velocity zones to suppress load swings. Then, we design a scheme to recalculate the amount of velocity and acceleration for traveling and traversing motions such that the reference trajectories return to the original final point during the decelerating zone with no load swing control (*K_θ_* = 0). This procedure is summarized in the following steps with an illustration for reference traveling trajectory as an example in Figure 5:

*Step 1*: Determine the correction velocities (*v_xrc_*, *v_yrc_*) needed for the trolley to move from its deviated reference position (*x_rd_*, *y_rd_*) occurs at the end of constant-velocity zone to the final desired location (*x_rf_*, *y_rf_*) within decelerating time (*t_b_* seconds) in parabolic form, i.e., *v_xrc_* = 2 (*x_rf_* − *x_rd_*)/*t_b_* and *v_yrc_* = 2 (*y_rf_* − *y_rd_*)/*t_b_*.
*Step 2*: Determine the correction reference trolley acceleration (*a_xrc_*, *a_yrc_*) needed for the velocities {*v_xrc_*, *v_yrc_*} to go to zero and set it in ***u****_c_xy_* with *K_θ_* = 0, i.e., *u_cx_* = *a_xrc_* = (*v_xrc_*/*t_b_*) and *u_cy_* = *a_yrc_* = (*v_yrc_*/*t_b_*) at time *t_f_*–*t_b_*.
*Step 3*: Set the initial conditions to [*x_rd_ v_xrc_*]*^T^* and [*y_rd_ v_yrc_*]*^T^* at time *t_f_*–*t_b_* for the traveling and traversing reference models in reference signal generator block, respectively.

The correction steps explained above would be sufficient when the amount of deviation in position is not significant. However, due to some unexpected rise in load swing, such as wind blow, large deviation in reference trajectories could occur, since load swing control would try to reduce load swing intensity by modifying reference accelerations considerably. Thus, correction velocities may exceed the maximum permissible velocity of the actuators if the updated trajectories happen to fall behind the original ones. In that case, the velocities can be set to their maximum values to protect the actuators, but the reference trolley position will not get to the original final destination within decelerating time and we would still have position error at the end.

**Figure 6:**
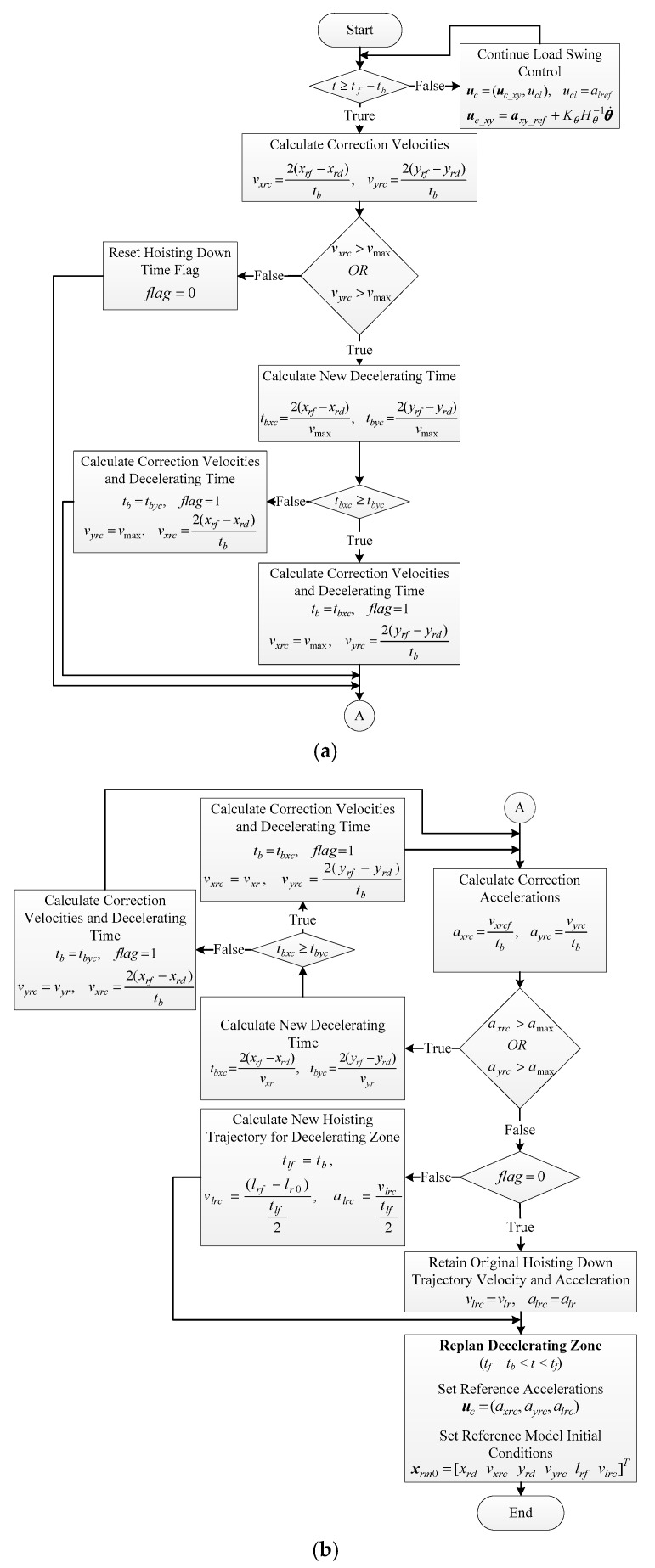
The proposed motion planning flowchart. (**a**) Calculation of correction velocities; (**b**) Calculation of correction acceleration, recalculation of correction velocities, hoisting velocity, and acceleration if decelerating time is extended.

In addition, it is possible that either of the correction accelerations become greater than the maximum admissible actuators acceleration, which can further complicate the situation. Thus, decelerating time has to be increased to allow the correction being conducted within permitted velocity and acceleration range. Moreover, if the decelerating time increases, the hoisting trajectory should be adapted to the new decelerating time. Therefore, there must be a procedure to determine the optimum decelerating time if the violation of constraints happens. This will guarantee the fastest feasible load transportation time with robust load swing suppression and safe control operation. The following flowchart in Figure 6 depicts re-planning of the decelerating zone parameters.

As can be seen in Figure 6a, the load swing control is active until reaching decelerating zone at *t* = *t_f_* − *t_b_* (the times are chosen to be an integer multiples of sampling time, i.e., *t* = *kT_s_* = *t_f_* − *t_b_*). Correction velocities are then calculated according to Step 1, and they are checked against maximum permissible velocity *v*_max_ using the logic operator OR (it is assumed that maximum permissible velocity is the same for both traveling and traversing motions). If none of the correction velocities is greater than *v*_max_, the correction accelerations will be calculated in Figure 6b and the hoisting down time flag is set to zero, showing that up to this stage there is no constraint violation. If any of the correction velocities is greater than *v*_max_, new decelerating times are calculated using *v*_max_ for both traveling and traversing motions (*t_bxc_*, *t_bxc_*). The final decelerating time is determined by the longer one, as it shows which direction requires more time to fix the deviation. New correction velocities are then calculated based on the new decelerating time, and the hoisting down flag is set to one showing that the hoisting trajectory needs to be updated as shown in Figure 6a.

Next, correction accelerations should be calculated as in Step 2. However, even if the decelerating time was extended in previous step, we would still need to make sure that the correction accelerations will not exceed the maximum admissible acceleration *a*_max_ with the new velocities and decelerating time. If the correction accelerations are less than *a*_max_, then it is just needed to check whether the decelerating time has extended in the previous step or not by checking the hoisting down time flag. If the flag is zero, it means that neither the correction velocities nor the corresponding correction accelerations were needed to be recalculated. Otherwise, if hoisting down time flag is one, the reference hoisting velocity and acceleration should be updated based on the new decelerating time, and then the reference trajectories for decelerating zone is re-planned based on Step 3.

However, if any of the correction accelerations turns out to be greater than *a*_max_, a different approach is used to guarantee the convergence of the algorithm. Therefore, regardless of whether the decelerating time has extended in the previous step, if one of the correction accelerations is greater than *a*_max_, the correction velocities are set back to their original normal velocities that had been designed for the reference traveling and traversing trajectories, i.e., *v_rx_* and *v_ry_*, respectively. The decelerating time for fixing the deviations is then recalculated using *v_rx_* and *v_ry_* which is longer than both the original decelerating time and the one calculated in the previous step (if that had occurred). In this way, the new correction accelerations are guaranteed to be less than *a*_max_, even less than the original accelerations, at the cost of extending decelerating time long enough to make sure none of the velocities and accelerations would exceed their maximums.

Therefore, the second check of the correction accelerations would always get to the hoisting down time flag checking, and ultimately, the whole procedure depicted in the flowchart in Figure 6 would end in maximum 15 cycles when decelerating zone is reached after constant-velocity zone. Hence, all we need do is to make sure that the processor frequency of the main controller responsible for executing control program is faster than 1/(15 × *T_s_*). This will guarantee that re-planning of the decelerating zone at *t_f_* − *t_b_* will be successfully finalized before the next sampling time.

## 6. Practical Results

### 6.1. Specifications of the 3D Overhead Crane

**Figure 7:**
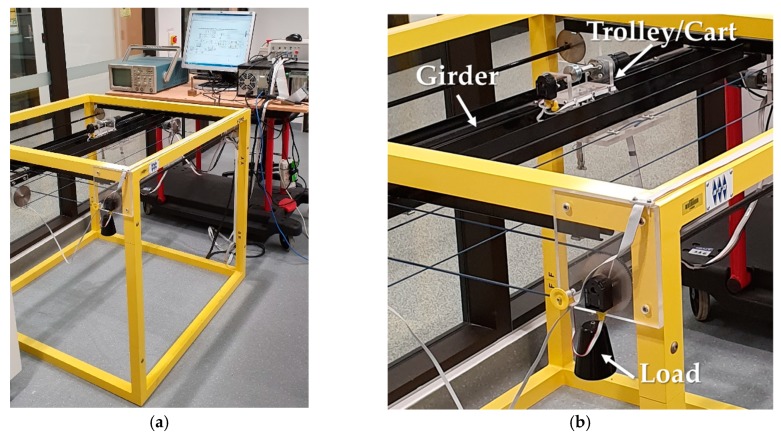
(**a**) The experimental overhead crane setup used in this study; (**b**) Zoomed-in view.

The practical device to run the tests and validate our control system design for in this work is manufactured by INTECO Limited [58] as shown in Figure 7. This setup is driven by three 24-volt PM DC motors. The measurements are made by five identical position encoders with the resolution of 4069 pulses/rotation for traveling and traversing positions, hoisting rope length, and swing angles in *X* and *Y* directions. The setup is equipped with RT-DAC/PCI9030 multipurpose digital I/O board connected to a power interface board and installed on a HP desktop computer with Intel^®^ Core2Due 3.00 GHz CPU with 3 GB RAM (Intel, Santa Clara, CA, USA). This setup works with the sampling time *T_s_* = 0.01 seconds and all functions of the board are accessible from a Real-Time Toolbox provided by the manufacturer that operates in MATLAB^®^ (MathWorks, Natick, MA, USA) and Simulink^®^ (MathWorks, Natick, MA, USA) software, all purchased by the university in Sydney, Australia.

### 6.2. Experimental Results and Validation

The designed control system is tested under three different scenarios where in each scenario, the reference trajectories are designed with low speed and fast speed, to compare the performance of the control system in handling high-speed load transportation while suppressing load swings, particularly with repetition to examine repeatability [53]. Table 1 provides the identified overhead crane parameters used in the controller design with known load mass of *m* = 0.8 kg and *g* = 9.81 m/s^2^ [53]. The reference trajectories are design in accordance with our proposed motion planning scheme in Section 5 and LSPB trajectory form as in Figure 4, in which the motion begins from a given initial location and ends at a given destination. After a waiting period to replicate the load swapping, the overhead carne transports the load back to initial location. The parameters for traveling, traversing and hoisting motions are provided in Table 2. The maximum permissible velocity and acceleration for traveling and traversing motions are given as *v*_max_ = 0.3 m/s and *a*_max_ = 0.2 m/s^2^ [58]. Table 3 shows the controller parameters where the observer gain *L* is calculated to make sure the state estimation dynamics are much faster than the overall the close-loop system. Supplementary materials including the source files and recorded data of the real-time experiments were provided during the submission phase of this article for verification of the results.

Load swing control (LSC) is turned off in the first scenario (Scenario I) as well as feedforward control (FFC) (i.e., LSC = Off, FFC = Off, which means *K_θ_* = 0 and ***u****_ff_* (*k*) = 0 where 0 denotes a zero vector with proper size). In the second scenario (Scenario II) feedforward control is tuned on only while load swing control is still deactivated (FFC = On, LSC = Off). In the final scenario (Scenario III) both feedforward control and load swing control are tuned on (LSC = On, FFC = On).

**Figure 8:**
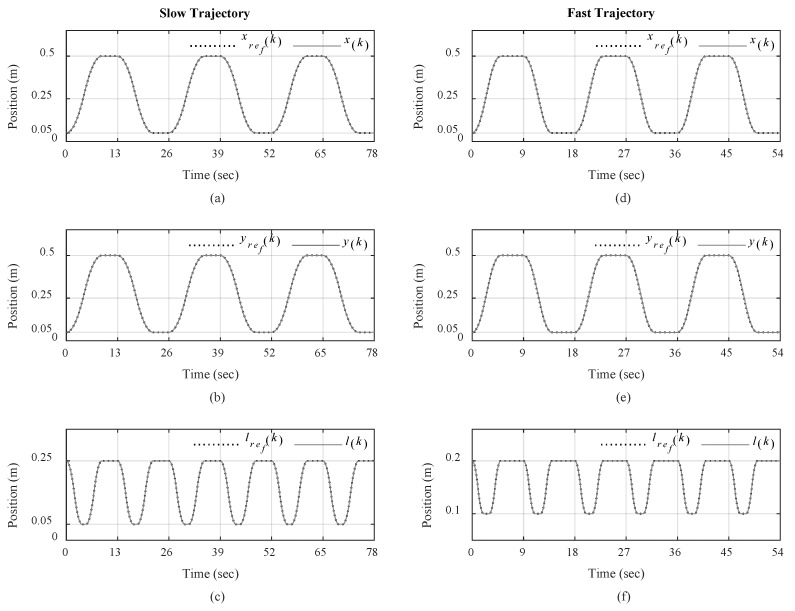
Actual and reference trajectories: (**a**) Traveling; (**b**) traversing, and (**c**) hoisting for slow trajectory; (**d**) Traveling; (**e**) traversing, and (**f**) hoisting for fast trajectory under Scenario III.

**Figure 9:**
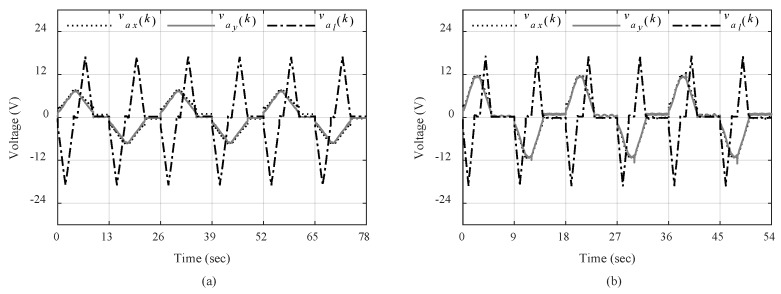
Control input voltages: (**a**) Slow trajectory; (**b**) Fast trajectory.

**Figure 10:**
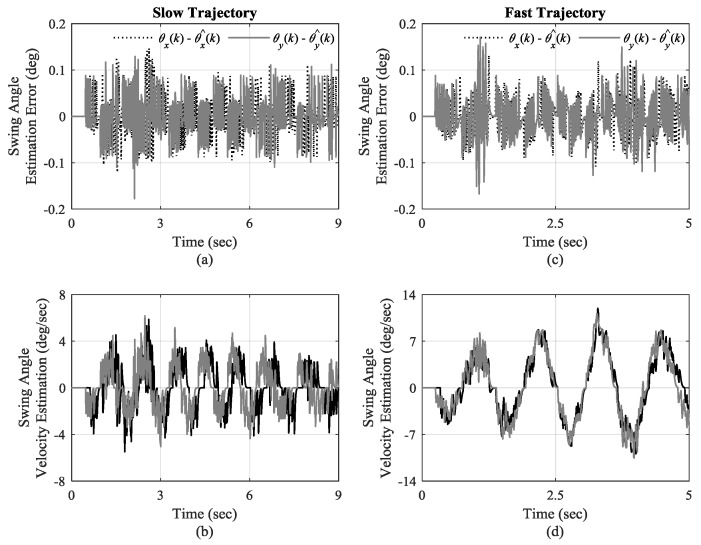
(**a**,**b**): Estimation error of swing angle and their velocities estimates for slow trajectory, respectively; (**c**,**d**): Estimation error of swing angle and their velocities estimates for fast trajectory, respectively.

**Figure 11:**
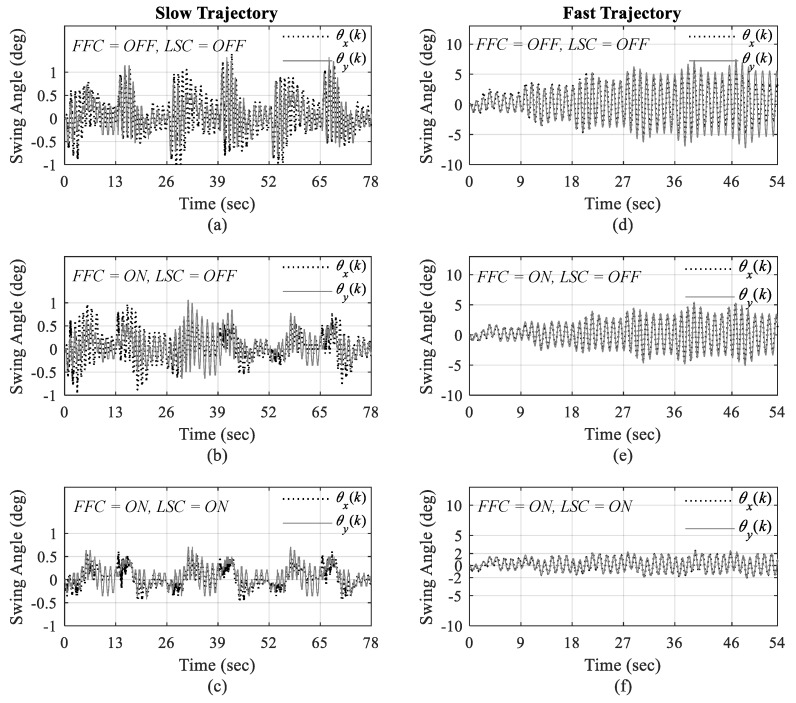
Load swing in *X* and *Y* directions for slow trajectory in (**a**) 1st; (**b**) 2nd; (**c**) 3rd Scenarios and for fast trajectory in (**d**) 1st; (**e**) 2nd, and (**f**) 3rd Scenarios.

**Figure 12:**
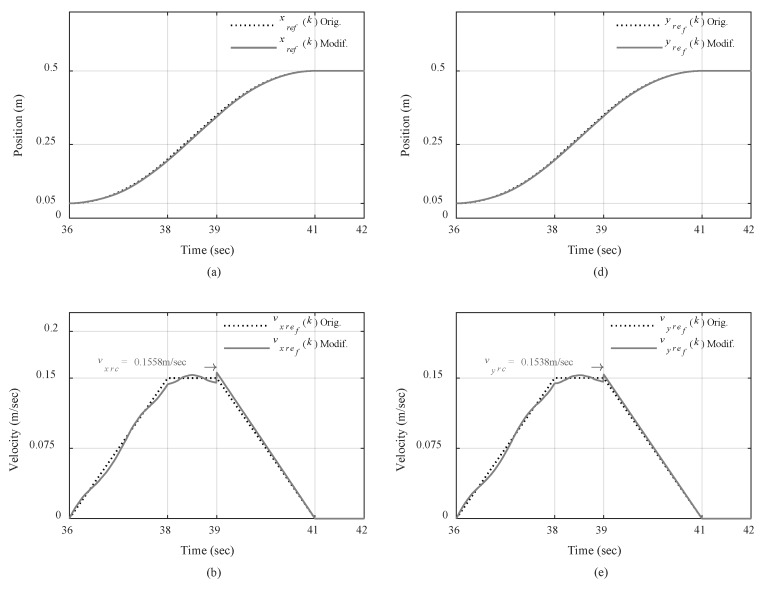

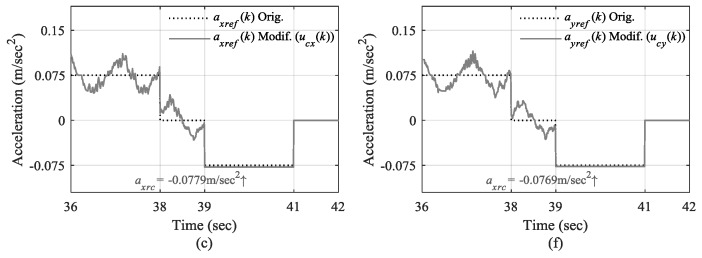
Original and modified reference trajectories in 3rd Scenario for fast trajectory: (**a**) Positions; (**b**) velocities; (**c**) accelerations for traveling motion; (**d**) Positions; (**e**) velocities, and (**f**) accelerations for traversing motion.

**Figure 13:**
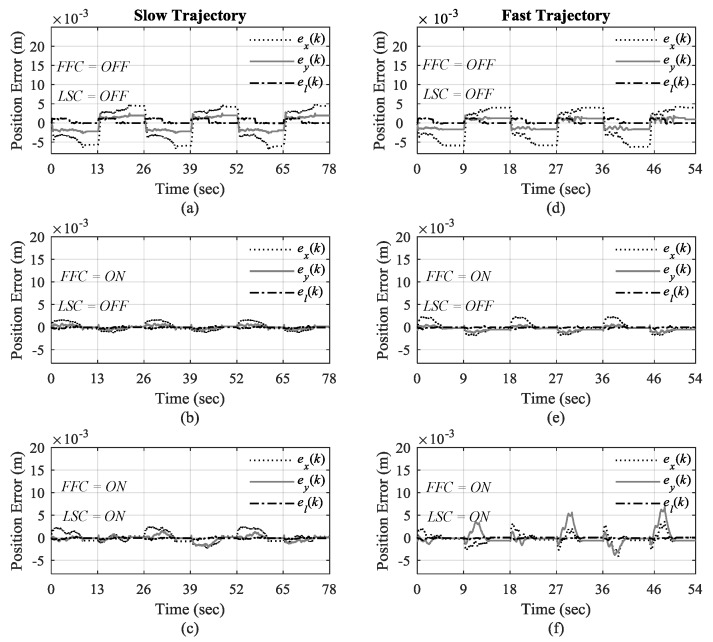
Trajectory tracking error for slow trajectory in (**a**) 1st; (**b**) 2nd; (**c**) 3rd scenarios, and for fast trajectory in (**d**) 1st, (**e**) 2nd; and (**f**) 3rd Scenarios.

These experiments will show how each part of the control system contributes to the control of overhead crane for automatic load transportation. The practical results are presented in Figure 8, Figure 9, Figure 10, Figure 11, Figure 12 and Figure 13. The trajectory tracking performances on each direction are shown in Figure 8 with slow-speed and fast-speed motions under Scenario III. As can be seen, the comparison between the actual trajectories and the reference ones indicates that the designed discrete-time control system can follow the reference trajectories with high performance and accuracy even in multiple repetitions. It can also be seen that the control system can handle high-speed load hoisting in both slow and fast trolley motions. Control input voltages are displayed in Figure 9 for both trajectories under the third scenario. It can be seen that all input voltages for PM DC motors are maintained within the nominal voltage range of ±24 V.

Optical encoders were used for angular position measurement of swing angles. To demonstrate the performance of the designed high-gain swing angle observer, swing angle estimation errors with the estimates of their velocities are plotted in Figure 10 for the first transition of the trajectories. As can be seen in Figure 10a,c, the designed swing angle observer can estimate swing angles with high accuracy in the range of ± 0.1 degree in both slow and fast motions. The estimates of swing angle velocities are given in Figure 10b,d. It is seen that swing angel velocities are estimated with less oscillations for fast trajectory in Figure 10b compared to the slow trajectory ones in Figure 10d, due to larger load swings in fast trajectory.

The measurements of load swings under three scenarios for both slow and fast trajectories are displayed in Figure 11. It can be seen that the largest load swings happens when neither load swing control nor feedforward control are turned on in Scenario I as illustrated in Figure 11a,d.

The effect of adding feedforward control in Scenario II can be seen in Figure 11b,e, which reduces the overall amount of load swing throughout the operation to some extent as we expected. However, with both load swing control and feedforward control in action, the amplitude of swing angles is significantly declined in the third scenario as illustrated in Figure 11c,f. This drop is quite noticeable in fast trajectory in Figure 11c, with around 60 percent reduction from maximum magnitude of 5 degrees in Scenario I and II, to about 2 degrees in Scenario III. These results show that the designed load swing control is able to suppress load swings as it is proven in the stability analysis of load swing given in Section 4.1.

The performance of reference signal generator that provides the modified reference trajectories through updating reference trolley accelerations using load swing control and our proposed motion planning scheme is pictured in Figure 12. The graphs show the comparison between the original reference trajectories and the modified ones in the third repetition of the fast trajectory for traveling and traversing motions, as an example, when the overhead crane was controlled under Scenario III.

The deviation in reference position trajectories, as shown in Figure 12a,d, may not be very visible due to the scale of figures. However, Figure 12c,f clearly depict how the modified reference accelerations differ from the original ones, and consequently affect the reference velocity profiles as illustrated in Figure 12b,e. Moreover, it can be seen that at the end of constant-velocity zone at 39th seconds, the decelerating zone re-planning procedure calculates the correction velocities (*v_rxc_*, *v_ryc_*) in Figure 12b,e and the correction accelerations (*a_rxc_*, *a_ryc_*) in Figure 12c,f, such that the reference position profiles can return to the original final values at the end of decelerating zone as is showed in Figure 12a,d. Thus, it is guaranteed that the load will be transported to the intended final destination knowing that the designed control system can successfully track the reference trajectories for traveling, traversing, and hoisting motions without violating the actuators’ constraints.

The trajectory tracking errors are demonstrated in Figure 13 under the aforementioned three scenarios for slow and fast trajectories. As expected, the positions in *X*, *Y*, and *Z* directions have the highest error in Scenario I with no load swing control, and also no feedforward control to compensate for load disturbances as shown in Figure 13a in slow trajectory, and more noticeable in Figure 13d in fast trajectory. Since the disturbances are intensified when the overhead crane moves with high speed, the tracking error is much higher without any compensation measure.

In the second and third scenarios where feedforward control is active, the performance of load positioning is improved considerably, particularly at the end of each transition with the tracking error less than ± 1 millimeter, for both slow and fast trajectory in Figure 13b–f. Not surprisingly, due to using load swing control in Scenario III, the tracking error increases during accelerating and constant-velocity zones in each transition of the trajectories in Figure 13f compared to Scenario II in Figure 13e for fast trajectory. For slow trajectory, however, the deterioration of tracking error is not considerable in the second and third scenarios, as shown in Figure 13b,c due to lower speed of the overhead crane motion. Nevertheless, the combination of load swing control and feedforward control creates a trade-off between suppressing load swings and maintaining a low tracking error to provide high-performance control operation, particularly at the destination, as well as effectively handling high-speed load hoisting.

## 7. Discussion

The other interesting future topic in this area, which is closely related to motion planning, is the problem of obstacle avoidance during each zone of the trajectory and how to update the reference trajectories such that it does not create undesired load swings, which has not been fully investigated in the literature [59].

## 8. Conclusions

In this work a new overhead crane control system has been developed in order to deliver high performance control operation in both reducing the load swings and transporting the load robustly and as fast as possible with less complexity in the design and implementation. The control system is designed discrete-time using state feedback control, which delivers accurate load positioning with robust load swing suppression and high-speed load hoisting capability. A new modelling approach has been developed for the 3D overhead crane which is adopted from robot manipulator control technique known as independent joint control, where the actuators of the overhead crane are considered for the design of the control system. The nonlinear equations of motion for the 3D overhead carne are regarded as measured disturbances in the model. Using computed torque control technique, a nonlinear feedforward control is designed to improve the trajectory tracking and reduce the impact of nonlinear couplings. The designed control system has four main parts where each part is in charge of once aspect of the control system operation: A state feedback controller designed for trajectory tracking, a reference model which works in conjunction with a new motion planning scheme for optimum operation, a feedforward controller to deal with reducing the influence of nonlinear disturbances, and finally, a new load swing control which updates the reference traveling and traversing accelerations in real time to guarantee load swing suppression. The new motion planning scheme has been designed to provide minimum-time reference trajectories by taking into account maximum permissible velocities and accelerations of actuators as well as allowing suppression of load swing during control operation in real time. The robustness and stability of the designed control system in discrete time have been verified analytically and experimentally with several practical tests. The results showed that the designed control system can deliver high performance control operation applicable to both 2D and 3D overhead cranes with the ability of high-speed load hosting. Finally, A video of the control operation based comparing the proposed control system design in full action compared with only position control on the 2D overhead crane is available online on YouTube (click https://www.youtube.com/watch?v=vD_S3uwvOsA).

## Figures and Tables

**Table 1:** Experimental Overhead Crane Setup Parameters.

Parameters for *i = x,y,z*	*J_ei_* (kg/m)	*B_ei_* (N.s)	*r_gi_*	*R_pi_* (m)	*K_ei_* (N·m/A·Ω)	*α*_1*i*_	*α*_2*i*_
Traveling	75 × 10^−4^	96.3 × 10^−3^	13 × 10^−3^	37.5 × 10^−3^	14 × 10^−4^	23 × 10^−4^	21 × 10^−4^
Traversing	40 × 10^−4^	97.5 × 10^−3^	13 × 10^−3^	37.5 × 10^−3^	14 × 10^−4^	14 × 10^−4^	11 × 10^−4^
Hoisting	66 × 10^−4^	24.5 × 10^−2^	13 × 10^−3^	13.5 × 10^−3^	14 × 10^−4^	13 × 10^−4^	14 × 10^−4^

**Table 2:** Reference Trajectory Parameters.

Parameters (*x_ref_*, *y_ref_*)	(*a_xr_*, *a_yr_*) (m/s^2^)	(*v_xr_*, *v_yr_*) (m/s)	(*x_r_*_0_, *y_r_*_0_) (m)	(*x_rf_*, *y_rf_*) (m)	*t_b_* (s)	*t_f_* (s)
Slow motion	22.5 × 10^−3^	9 × 10^−2^	5 × 10^−2^	50 × 10^−2^	4	9
Fast motion	75 × 10^−3^	15 × 10^−2^	5 × 10^−2^	50 × 10^−2^	2	5
**Parameters** **(l_ref_)**	***a_lr_*** **(m/s^2^)**	***v_lr_*** **(m/s)**	***l_r0_*** **(m)**	***l_rf_*** **(m)**	***t_b_*** **(s)**	***t_f_*** **(s)**
Slow motion	50 × 10^−3^	10 × 10^−2^	25 × 10^−2^	5 × 10^−2^	4	9
Fast motion	100 × 10^−3^	10 × 10^−2^	2 × 10^−2^	10 × 10^−2^	2	5

**Table 3:** Proposed Discrete-Time Control System Parameters.

Parameters for *i = x,y,z*	$K_{i}$	$L_{i}$	$L_{\theta_{i}}$	$k_{\theta_{i}}$
Traveling	[12.9 × 10^2^ 1.1 × 10^2^]	[42.9 × 10^−2^ 26.5 × 10^−2^]*^T^*	[1 25]*^T^*	17 × 10^−2^
Traversing	[25.9 × 10^2^ 1.2 × 10^2^]	[41.5 × 10^−2^ 27.7 × 10^−2^]*^T^*	[1 25]*^T^*	17 × 10^−2^
Hoisting	[38.4 × 10^2^ 1.2 × 10^2^]	[43.5 × 10^−2^ 29.7 × 10^−2^]*^T^*	N/A	N/A

## Supplementary Materials

The following are available online at https://www.mdpi.com/1424-8220/19/15/3429/s1. The source files and data of the real-time experiments were provided to the reviewers during the submission phase of this article.
